# The role of complement in the pathogenesis of renal ischemia-reperfusion injury and fibrosis

**DOI:** 10.1186/1755-1536-7-16

**Published:** 2014-11-01

**Authors:** Juan S Danobeitia, Arjang Djamali, Luis A Fernandez

**Affiliations:** 1 Department of Surgery, Division of Transplantation, University of Wisconsin-Madison School of Medicine and Public Health, H4/782 Clinical Science Center, 600 Highland Avenue, 53792 Madison, WI, USA; 2 Department of Medicine, Division of Nephrology, University of Wisconsin- Madison School of Medicine and Public Health, UW Medical Foundation Centennial Building, 1685 Highland Avenue, 53705 Madison, WI, USA

## Abstract

The complement system is a major component of innate immunity and has been commonly identified as a central element in host defense, clearance of immune complexes, and tissue homeostasis. After ischemia-reperfusion injury (IRI), the complement system is activated by endogenous ligands that trigger proteolytic cleavage of complement components via the classical, lectin and/or alternative pathway. The result is the formation of terminal complement components C3a, C5a, and the membrane attack complex (C5b-9 or MAC), all of which play pivotal roles in the amplification of the inflammatory response, chemotaxis, neutrophil/monocyte recruitment and activation, and direct tubular cell injury. However, recent evidence suggests that complement activity transcends innate host defense and there is increasing data suggesting complement as a regulator in processes such as allo-immunity, stem cell differentiation, tissue repair, and progression to fibrosis. In this review, we discuss recent advances addressing the role of complement as a regulator of IRI and renal fibrosis after organ donation for transplantation. We will also briefly discuss currently approved therapies that target complement activity in kidney ischemia-reperfusion and transplantation.

## Review

### The complement system

The complement system consists of a family of circulating proteins, cell-surface receptors, proteolytic enzymes, and cleaved peptides that play an essential role in first-line host defense against pathogens and in the regulation of inflammation [1]. Complement activation is a tightly regulated process that requires sequential and organized activation of proteins in order to form the effector molecules involved in host defense, pathogen clearance, and modulation of the inflammatory response [2]. This intricate network of proteins can be activated by three distinct pathways: classical, lectin, and alternative, all of which converge in the formation of fraction C3 and ultimately in the downstream formation of the activation products, C3a, C3b, C5a, and the membrane attack complex (C5b-9). The classical pathway is triggered upon binding of antigen to surveillance proteins such as immunoglobulins (IgM or IgG) or C-reactive protein forming immune complexes that bind C1q. In turn, C1q activates fractions C1r and C1s, which are ultimately responsible for cleaving C4 and forming the C3 convertase. The lectin pathway is activated by the binding of complex carbohydrate residues commonly found on the surface of pathogens to circulating mannose-binding lectin (MBL) or ficolins. Both MBL and ficolins circulate in association with MBL-associated proteins (MASPs) which, upon activation, allow auto-activation and formation of MASP2, the protein in charge of cleaving fraction C4 in the lectin pathway. As in the classical pathway, C4 cleaves C2 forming the C3 convertase (C4bC2a). The alternative pathway is activated by direct binding of hydrolyzed C3b to the surface of bacterial membranes.

**Figure 1:**
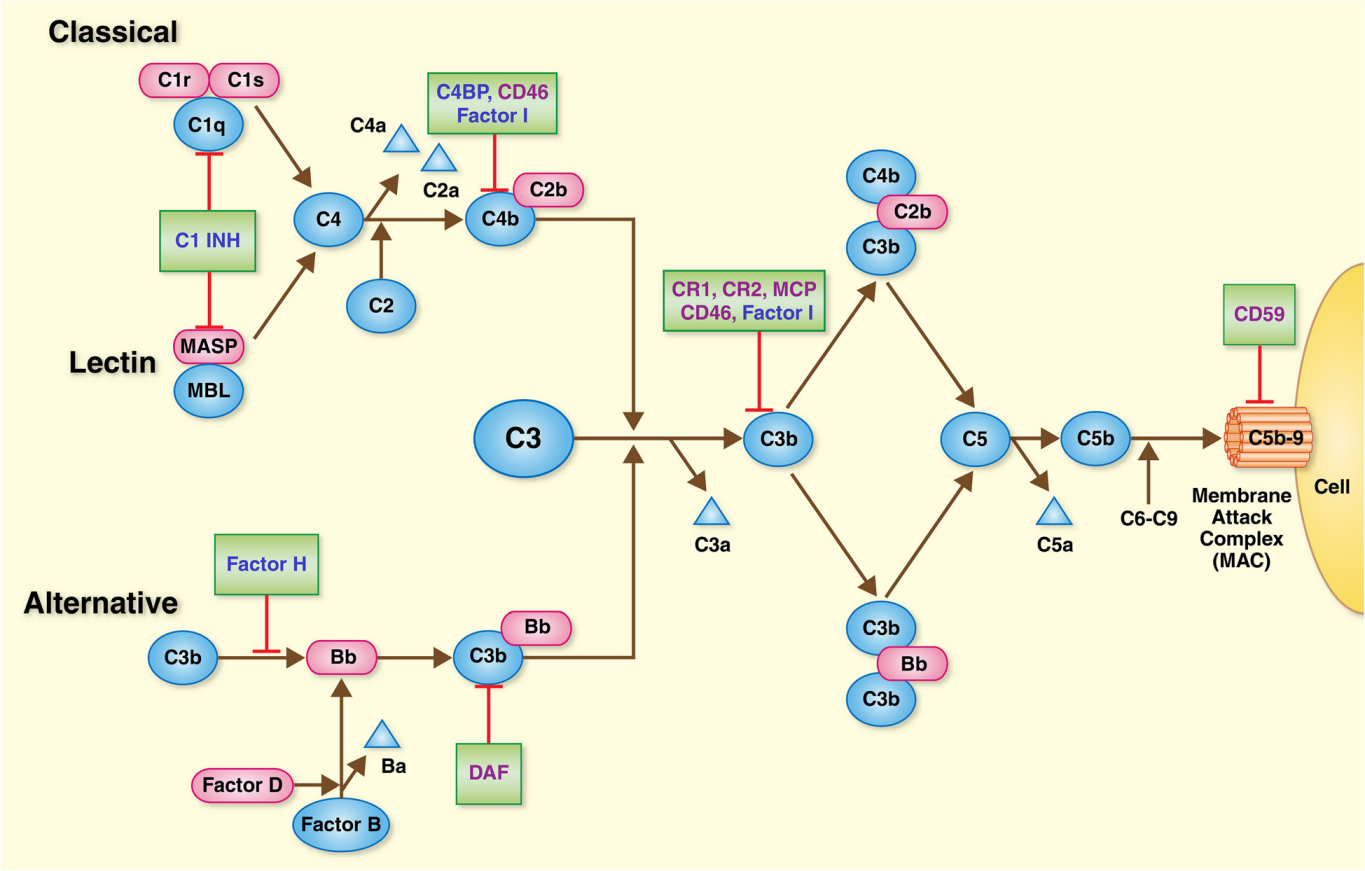
**Overview of the complement system.** Activation of the complement system by the classical, lectin, and alternative pathways results in cleavage of the C3 and C5 fractions by the C3 and C5 convertases, which, in turn, generate the opsonin C3b, anaphylatoxins C3a and C5a, and the membrane-attack complex (MAC; C5b–C9). Regulation of complement activation and deposition is controlled by fluid-phase (C4BP, Factor H, and C1-INH) and membrane-bound regulators (CD35, CD46, CD55, and CD59).

In addition to the proteins involved in cleavage and activation of the complement cascade, the complement system is also composed of a series of soluble (C4BP, Factor H, and C1-INH) and membrane-bound (CD35, CD46, CD55, and CD59) regulatory proteins that prevent excessive activation and consumption of complement components [3]. These regulators control complement activation mainly by serving as co-factors for Factor I in the proteolysis of the C3a and C5a convertases or by directly accelerating the decay of both of these convertases. Complement receptor 1 (CR1, CD35) is found on the surface of erythrocytes, neutrophils, dendritic cells, and T and B lymphocytes, and controls complement activation by serving as a cofactor for Factor I and by direct inhibition of classical and alternative pathway convertases. Likewise, CD46 (MCP) has a dual role serving as a cofactor for Factor I and promoting C3 degradation while CD55 (decay-accelerating factor) has only been shown to accelerate C3 convertase decay and CD59 (Protectin) functions by binding to complex C5b-8 and inhibiting membrane attack complex (MAC or C5b-9) assembly [3]. The soluble regulators C4BP and Factor H exert their regulatory function by serving as cofactors for Factor I and accelerating convertase decay [4,5]. Finally, circulating C1 inhibitor (C1-INH) is a serine protease inhibitor that inactivates proteases C1r, C1s, and MASP1 and 2 in the complement system preventing mainly the activation of the cascade via the classical and lectin pathways, although recent evidence suggests it may have inhibitory properties over the alternative pathway as well [6] (Figure 1).

### Role of complement in renal ischemia-reperfusion injury (IRI)

Ischemia-reperfusion injury (IRI) is a common mechanism of injury in a wide variety of conditions characterized by limited tissue perfusion. During the ischemic period, tissues are deprived of oxygen and nutrients required to maintain normal metabolism and energy homeostasis. As a result, cells in ischemic tissues become necrotic and release a variety of endogenous ligands known to stimulate innate immune responses [7]. Upon restoration of perfusion, endogenous ligands from necrotic and apoptotic cells activate incoming innate immune cells and exacerbate the inflammatory tissue and organ injury [8]. IRI has been consistently shown to promote quick recruitment and activation of neutrophils and macrophages to the injury site. Activated neutrophils migrate from peripheral circulation into the injured site where they become activated and release pro-inflammatory cytokines, chemokines, and reactive oxygen species (ROS), both locally and systemically, which have been shown to play a pivotal role in cell apoptosis and necrosis [9,10].

The complement system has been strongly associated with the inflammatory response to IRI [11-13]. Although initially it was believed that the complement system was exclusively involved in responses to non-self-antigens, recent research has provided a novel perspective into its intricate role in the sterile immune response to injury and tissue repair. Following IRI, the release of danger-associated molecular patterns (DAMPs), neo-antigen formation, and immune complex formation can activate the complement system by any of the three main pathways [14]. Using animal models of IRI, it has been demonstrated that complement can be differentially activated depending on the organ system being affected and, in some cases, a combination of mechanisms of activation has been observed. It is well established that generation of C3 by all three pathways plays an important role in renal ischemic injury and it is accepted that formation of the MAC (C5b–C9) induces direct cell lysis and tubulointerstitial injury [15]. Traditionally, the classical pathway has been implicated in the pathogenesis of complement driven IRI [16]. However, studies in rodent models of renal IRI indicate a predominant role for the alternative pathway and the classical pathway does not appear to impact reperfusion injury when tested in C4-deficient mice or RAG-1-deficient animals which are unable to generate IgM or IgG [17,18]. On the other hand, Factor B-deficient mice known to be defective in alternative pathway activation, show a marked reduction in functional and morphological injury induced by ischemia and reperfusion [19]. More recently, the lectin pathway has gained attention and has been shown to play a pivotal role in the pathophysiology of ischemic kidney damage [20]. Mice deficient in MBL-A and MBL-C are protected from renal IRI and reconstitution of mutant animals with recombinant MBL led to injury levels comparable to those of wild-type mice used in the study [21]. Furthermore, animals lacking MASP2 are protected from injury following both myocardial and intestinal ischemic injury and a report by van der Pol in a rat model of renal IRI suggests a novel role for MBL in cellular injury independent of complement activation in which internalization of circulating MBL resulted in direct tubular necrosis [22,23]. Taken together, these studies suggest an emerging and important role for the lectin pathway mediating ischemic kidney damage. However, findings in rodent models of IRI are not in complete agreement with those from larger animal models and humans, in which complement activation in the context of IRI appears to behave differently. These studies suggest that activation of complement by the classical pathway and lectin pathway have detrimental effects following renal ischemic injury. Recently, Castellano et al. reported that inhibition of classical and lectin pathways through the use of recombinant human C1 inhibitor (rhC1INH) resulted in attenuated renal dysfunction in a pig model of IRI [24]. Clinically, the role of the lectin pathway in IRI and transplant injury remains controversial. Berger et al. analyzed a cohort of renal transplant patients and found that high pre-transplant MBL correlated with the severity of rejection and the rate of allograft loss [25]. However, analysis of lectin gene profiles of kidney donors and recipients failed to find associations between MBL and MASP2 genotypes with transplant outcome and a recent study by Bay et al. suggests poor graft survival in non-HLA immunized kidney recipients with low MBL serum levels [26,27].

To add complexity to the process, complement activation mechanisms also vary according to different organ systems. Using models of IRI, it is apparent that liver injury is mediated primarily by classical complement activation [28,29], whereas myocardial and intestinal reperfusion injury require both classical and lectin pathways to mediate pathological damage [22,30-32]. Such discrepancy in the findings between animal and human models, different organ systems, and the conflicting results from clinical studies, indicate a diverse role for complement in IRI. Some of the most interesting products of complement activation are the cleaved products C3a and C5a, also termed anaphylatoxins. These potent pro-inflammatory peptides interact with G-protein-coupled receptors on the surface of immune and non-immune cells and promote the activation inflammatory leukocytes, chemotaxis, histamine release, and increased vascular permeability [33]. In addition, recent evidence indicates an important role in antigen presentation to naïve T cells and in the activation and regulation of alloimmune responses [34-36].

**Figure 2:**
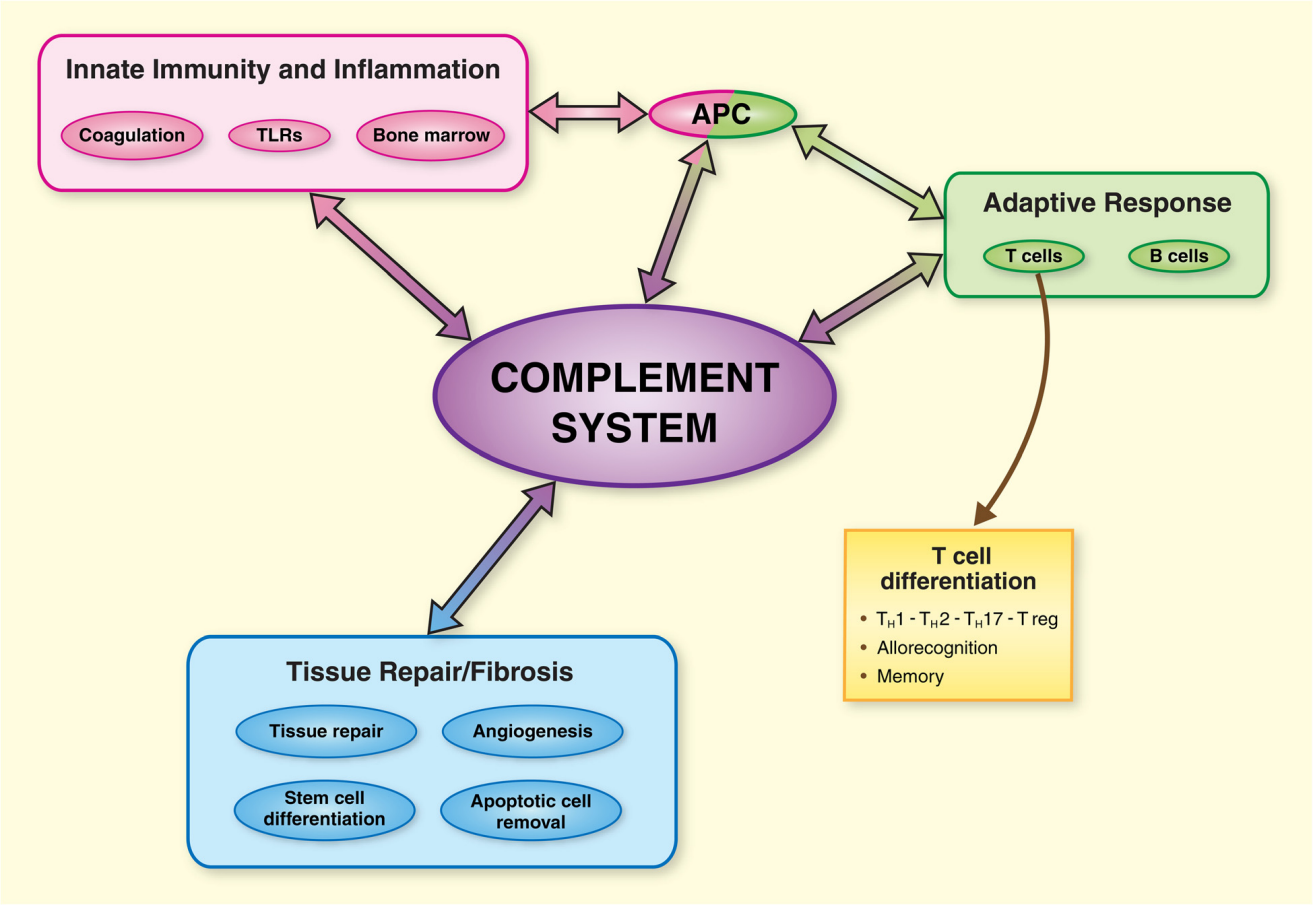
**Multifaceted activity of the complement system in immunity.** Classically, the complement system has been considered a component of innate immunity with limited activity outside the mechanisms that govern host defense. However, in recent years we have observed an ever expanding role for complement in the regulation, orchestration, and amplification of the immune response by regulating innate immunity, adaptive responses, and tissue repair.

In the mouse kidney, both resident innate immune cells as well as parenchymal tubular cells express receptors for C3a (C3aR) and C5a (C5aR) [37,38]. A recent study by Peng et al. provides compelling evidence of the deleterious role of C5aR, and to a lesser extent C3aR, signaling in renal IRI [37]. Mice harboring C5aR or combined C3aR/C5aR deficiency were protected from ischemic injury at 24 and 48 hours post-reperfusion and showed significant reductions in BUN serum levels, pro-inflammatory cytokine and chemokine mRNA expression, and tissue infiltration by activated immunocytes. Moreover, they showed that tubular epithelial cells and macrophages cultured under hypoxia/reoxygenation conditions respond to C3a and C5a stimulation by expressing cytokines, such as IL-6, TNF-α, KC (IL-8), and KIM-1, suggesting this to be a central mechanism involved in the process of early neutrophil recruitment, degranulation, and tissue inflammation in the post-ischemic period. These observations are supported by earlier studies in which the use of either C5a receptor antagonist or siRNA silencing of the C5aR was effective in attenuating renal damage and down-regulating the inflammatory response in rodent models of IRI [38-40] (Figure 2).

Animal models of IRI constitute a practical approach to the study of transplant reperfusion. However, renal IRI provides a limited approach in the study of transplant reperfusion injury as many factors play a role in the complex interplay of graft acceptance and rejection. As an example, in models of IRI, the contribution of T and B cell responses to allogeneic tissue are overlooked. In this context, the complement system is involved in the pathogenesis of acute and chronic rejection leading to graft loss in the setting of antibody-mediated rejection (AMR) and formation of donor specific antibodies (DSA) that trigger the complement cascade. Complement activation has been shown to correlate with DSA levels as indicated by C4d deposition in the peri-tubular capillaries. Mechanistically, HLA present on the allograft endothelial cell is bound by DSAs and activate the C1 complex. Downstream cleavage of C1 leads to cleavage of C4 forming C4d, which then binds covalently to vascular endothelium in the renal allograft and provides a marker for complement identification by immunohistochemistry [41]. Another possible mechanism of DSA-induced acute allograft damage may involve direct tubular injury by MAC; recent evidence suggests that the use of Eculizumab decreases the incidence of acute AMR in sensitized renal transplant recipients, suggesting an important role for C5a formation in this process [42]. In chronic AMR, complement activation requires deposition of DSA and the presence of donor-specific HLA antibodies correlates well with outcomes after kidney transplantation [43]. Subsequent activation of complement leads to C3a and C5a formation and, as described above, these anaphylatoxins promote activation and recruitment of inflammatory cells into the graft [44]. Chronic stimulation by migrating activated protein C is likely to induce cellular injury in response to continuous signaling by infiltrating inflammatory immunocytes and from the sustained expression of cytokines, chemokines, and pro-thrombotic/pro-fibrogenic factors [45]. However, chronic AMR may also be primarily complement independent in the absence of C4d deposition by activation of DSAs that could lead to progressive glomerular injury and transplant glomerulopathy.

### Complement activation in the brain-dead organ donor

Brain death is the irreversible loss of function of all parts of the brain, including the brain stem. Loss of brain stem function and the autonomic storm that follows catastrophic brain injury have been associated with tissue hypoperfusion, dysregulated metabolism, and generalized inflammation, all detrimental to organ quality and function [46-48]. In recent years, several reports have highlighted an emerging role for complement in the pathogenesis of tissue injury in the context of organ donation [49]. Early studies by Kusaka et al. indicate that as early as 1 hour post brain death induction, C3 is detectable in the glomeruli and vascular endothelium of brain-dead rats [50]. This increased expression of renal C3 has been widely associated with poor allograft function and an increased rate of cellular rejection in animal models of kidney transplantation [51-53]. Furthermore, using a mouse model of brain-death, Atkinson et al. showed that C3 deficient animals had a significant decrease in cardiac troponin levels, reduced immunocytic infiltration to the heart, and markedly reduced expression of pro-inflammatory cytokines and chemokines compared to wild-type controls [54]. More recent evidence suggests that inhibition of complement activation in rodent models of brain-death is effective in protecting renal and cardiac grafts from inflammatory injury and prolonging graft and animal survival in the post-transplant period [54,55].

In human deceased donors, there is substantial evidence of complement activation in the pre-transplant period. A study comparing whole genome expression found a significant up-regulation of complement-related genes (C1q, C1s, C1r, C2, C3, C4, CFB, and CR1) in kidneys from deceased compared to those recovered from living donors. Overexpression of complement components in brain-dead donors correlated directly with length of cold-ischemia time and inversely with early and late graft function [56]. In a similar study, Damman et al. reported that high levels of the terminal MAC in the plasma of brain-dead donors was strongly associated with acute rejection within the first year post-transplantation [57]. Additionally, activation of the C5a-C5aR axis in kidney donors plays an important role in the amplification of the inflammatory response to brain death [58].

Complement activation has also been suggested as a potential tool to determine the degree of donor injury and predict graft function after transplantation. Elevated donor MBL levels have been associated with suboptimal graft function after transplantation [25]. Furthermore, a recent study analyzing 75 kidney transplant recipients showed that increased levels of circulating MAC (C5b-9) in the peri-transplant period and early after reperfusion, strongly correlated with poor allograft function and suggested the use of MAC as a clinical marker in the prediction of delayed graft function (DGF) [59]. In a similar study, de Vries et al. detected a transient release of soluble MAC in post-reperfusion samples from deceased donor kidney grafts but not from those obtained from living donors. However, sMAC release did not correlate to C5b-9 deposition in biopsied kidney grafts analyzed 45 min after reperfusion [60]. Collectively, both animal and human studies highlight the importance of pre-transplant activation of the complement system in the pathogenesis of tissue damage after brain death and suggest potential therapeutic targets for the improvement of organ quality and function.

### Complement system and the progression to fibrosis

The complement system has been linked to a wide variety of non-immunological processes, including modulation of stem cell biology, tissue regeneration, and progression to tissue fibrosis after injury [61-64]. There is ample evidence suggesting a role for complement activation in experimental fibrosis and repair. Using a model of liver regeneration, Strey et al. clearly showed how C3a and C5a are pivotal to the early priming stages of hepatocyte regeneration and a more recent report from DeAngelis confirms these observations and expands by showing a regulatory feedback mechanism involving NK cells, complement, and IL-4 modulating liver repair [65,66]. Using a similar approach, He et al. evidenced an effect of low-dose CR2-Crry in liver protection and enhanced regeneration by controlling IL-6 expression and STAT3 activation, reduced hepatic ATP depletion, and attenuated oxidative stress [67].

In the kidney, alterations in complement activation have been implicated in multiple disease processes leading to renal fibrosis, such as polycystic kidney disease, glomerulonephritis, hemolytic uremic syndrome, and renal transplantation [68-71]. However, the role of complement activation in the modulation of immunity and pathogenesis of renal fibrosis in the context of IRI remains a work in progress. IRI of the kidney is a well-established cause of renal fibrosis [72]. Factors such as sustained innate immune activation, endothelial cell dysfunction, hypoxia, and chronic microvascular injury have all been implicated in the maladaptive response that results in fibrogenesis and progression to chronic kidney disease [73-75]. Both complement activation and endothelial cell activation are hallmarks of IRI, DGF, and allograft injury early after kidney transplantation. IRI is an inflammatory process initiated at the endothelial surface of the vasculature and is associated with increased susceptibility to subsequent acute rejection episodes and the vascular changes associated with chronic rejection [76]. Experimentally, C5a has been shown to induce P-selectin expression and induction of neutrophil rolling upon binding to C5a receptors (C5aR) on the surface of endothelial cells (EC) and polymorphonuclear neutrophils (PMNs) [77]. This is followed by release of reactive oxygen species (ROS) which further amplify endothelial injury, endothelial gap formation with leak of plasma, inflammatory cell migration, and NF-κB translocation.

Using a mouse model of unilateral ureteral obstruction, Boor et al. showed that C5-knockout animals and animals treated with C5aR antagonist had significantly reduced tissue fibrosis at 5 and 10 days post-injury [78]. This protective effect appeared to be mediated by a reduction in C5aR-driven TGF-beta production suggesting a new role for anaphylatoxin C5a in the complex mechanisms involved in tissue repair. In a similar study, Bao et al. reported a significant reduction in inflammation and renal fibrosis after transplanting kidneys from Crry(-/-)C3(-/-) mice into C3aR and/or C5aR-knockout recipients [79]. They found that in this model it was C3aR deficiency which resulted in the observed protective effect and that the improved renal function and reduced fibrosis levels were the result of inhibited C3a-driven inflammatory injury rather than a direct profibrotic effect of C3a on injured tubular cells.

Although the link between inflammation and fibrosis is well established, the origin and mechanism of activation/differentiation of renal fibroblasts is still a matter of debate. Differentiation of tubular epithelial cells into mesenchymal cells (epithelial to mesenchymal transition) and the functional and phenotypical progression of endothelium to mesenchymal cells (endothelial to mesenchymal transition; EndMT) have been proposed as potential sources of myofibroblasts responsible for renal fibrosis after injury. Further, it has been proposed that endothelial cells which progress to EndMT may play a pivotal role in the early pathogenesis of renal fibrogenesis [80]. To test this hypothesis, Basile et al. used a rodent model of IRI and documented a significant loss of vascular density following injury, which was associated with interstitial expansion of endothelial cells expressing mesenchymal cell markers and suggesting endothelial to mesenchymal transition post-AKI [81]. In agreement with these findings, Curci et al. found a significant reduction in renal fibrosis in pigs receiving C1 inhibitor therapy prior to reperfusion injury. This study postulates that the anti-fibrotic effect of C1-INH therapy is related to reduction in Akt signaling within injured endothelium which led to inhibition of EndMT and prevented vascular rarefaction in injured kidneys [82]. Altogether, these studies highlight a central role for endothelium in the progression to fibrosis and a novel role for complement in the modulation of endothelial cell activation and EndMT. In addition, there is also experimental evidence suggesting complement activation can also induce fibrosis by direct modulation of fibroblast function. Cleavage of C3 and exogenous supplementation of C3a has been shown to induce renal human mesangial cells to convert to the synthetic phenotype by increasing the expression of osteopontin, matrix Gla, and collagen type 1 alpha 1 (collagen IV) mRNA [83]. Also, MAC formation is a promoter of peritubular myofibroblast accumulation and plays a role in the pathogenesis of renal fibrosis in various glomerulopathies [84].

**Figure 3:**
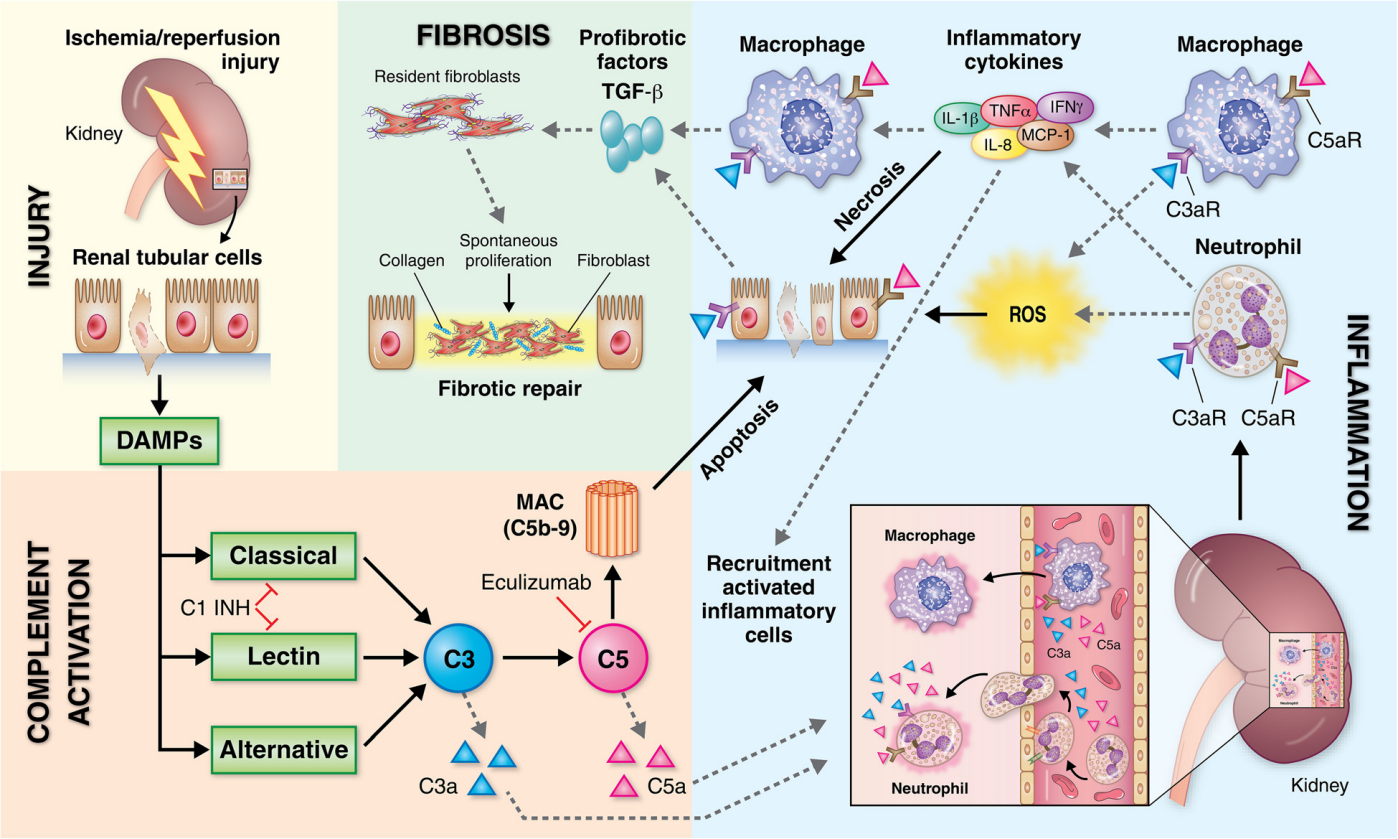
**Role of complement in renal ischemia-reperfusion injury, inflammation, and progression to kidney fibrosis.** Ischemia-reperfusion injury activates the complement system by release of endogenous ligands (DAMPs) from acutely injured tissue. The formation of the membrane attack complex (MAC) results in direct injury to the kidney by inducing apoptosis in epithelial tubular cells. In addition, the cleavage of C3 and C5 and subsequent release of anaphylatoxins (C3a and C5a) promotes inflammatory cell recruitment and release of pro-inflammatory cytokines/chemokines and reactive oxygen species, intensifying the immune response and further amplifying the level of tubular necrosis and apoptosis. Activated endothelium, monocytes and injured tubular epithelium have all been shown to secrete pro-fibrogenic factors such as TGF-β and PDGF in response to C3aR and C5aR ligation by C3a and C5a, respectively, which in turn activates local fibroblasts inducing collagen deposition and tissue repair. Dysregulated activation of complement and the subsequent inflammatory response ultimately results in maladaptive tissue repair and fibrosis.

Macrophage phenotype and function are critical determinants of fibrotic scarring and resolution of renal injury [85]. Monocytes from circulation that enter the kidney in response to inflammation undergo separate pathways of differentiation into classically activated M1 macrophages or the alternative M2 phenotype. Activation of M1 inflammatory macrophages may lead to the expression of MHC class II antigens and release of proinflammatory cytokines, further propagating inflammation and activating profibrotic pathways. In contrast, M2 macrophages secrete regenerative trophic factors that promote cell proliferation, reduce apoptosis, and stimulate angiogenesis. In murine models of IRI, M1 macrophages accumulate early in the first hours of reperfusion, through CCR2- and CX3CR1-dependent mechanisms, and produce IL-1α, IL-6, IL-12, and TNF-α [86,87]. Complement activation modulates macrophage differentiation and engagement of C5a-C5aR axis has been shown to promote M1 polarization and progression to inflammatory injury and fibrosis [88]. Moreover, in the kidney, generation of ROS is mediated by the NADPH oxidases (NOX) present in infiltrating PMNs and activated endothelium. Originally named gp91phox, Nox2 is the classical phagocytic NADPH oxidase, an enzyme that is naturally involved in the immune response including the “oxidative burst” [89,90]. It is one of the seven currently known Nox isoforms. Complement has been linked to oxidative stress and PMN activation in models of sepsis and inhibition of complement activation resulted in reduced ROS production and attenuation of PMN migration and activation to the site of injury [91,92]. We have demonstrated that Nox2 is an important mediator of renal fibrosis in kidneys undergoing chronic rejection and chronic cyclosporine induced nephrotoxicity [93,94]. Altogether, complement is likely to play a central role in the mechanisms leading to renal fibrosis by preventing inflammatory injury, activation and differentiation of inflammatory leukocytes, reducing the production of ROS and, most importantly, by preventing EndMT following acute ischemic injury. However, more work is required to fully elucidate the specific role of complement activation in the control of fibrotic responses and the progression to chronic kidney disease (Figure 3).

### Therapies targeting the complement system

Treatment aimed at blocking or attenuating complement activation during IRI and organ transplantation has gained increasing attention over the last two decades. Many molecules with the ability to control upstream complement activation and specific inhibitors of the terminal end-products of complement activation have been explored with varying degrees of success both in animal models and in the clinical setting. Here, we review two complement inhibitors currently approved by the food and drug administration (FDA) for the treatment of human disease with proven evidence of effectiveness in IRI and potential uses in the prevention of fibrosis and chronic tissue injury.

### C1 esterase inhibitor (C1-INH)

C1-INH is a soluble regulator belonging to the family of serin-protease inhibitors (serpins). Target proteases such as C1r, C1s, MASP1 and 2 (complement system), Factor XII and plasma kallikrein (contact system), Factor XI and thrombin (coagulation system) recognize the reactive center loop on the C1-INH and, upon cleavage, both molecules become covalently bound and the target protein is irreversibly inhibited [95]. These diverse regulatory effects on the fibrinolytic, contact, coagulation, and complement system make it an ideal candidate for treatment of IRI, antibody-mediated rejection and hyper-acute rejection after transplantation, and in the progression to fibrosis following acute kidney injury. A recombinant form of C1-INH (rhC1INH) has been approved for the treatment of hereditary angioedema [96]. Numerous pre-clinical studies have demonstrated effectiveness in the prevention of delayed graft function after ischemic injury in pigs and attenuated reperfusion injury in rodent models of intestinal, myocardial, hepatic, and neurological injury [24,97-100]. Moreover, pre-treatment with rhC1INH was shown to prevent the development of fibrosis in pigs subjected to renal IRI [82]. For the treatment of acute AMR, rhC1INH had a beneficial effect when tested in a kidney transplant model using presensitized baboons as recipients [101]. For lung transplantation, C1-INH therapy was recently evaluated in lung transplant recipients exhibiting early signs of primary graft dysfunction (PGD). In this study, C1-INH treatment improved the 1-year survival and reduced length of intensive care unit stay when compared to patients with early signs of PGD not receiving treatment outside the standard of care [102].

### Eculizumab

Eculizumab is a humanized monoclonal antibody that inhibits complement fraction C5 preventing formation of terminal activation products C5a and MAC (C5b-9). Eculizumab has been approved by the FDA for the treatment of paroxysmal nocturnal hemoglobinuria and atypical hemolytic uremic syndrome (aHUS). However, it has received major interest in the field of transplantation for the treatment of AMR and prophylactically in the prevention of post-transplant aHUS in kidney transplant recipients. A recent review summarizes the use of eculizumab for the prevention AMR and aHUS in kidney transplantation [103].

As described earlier, anaphylatoxin C5a and signaling through the C5aR play pivotal roles in mediating IRI and the dysregulated inflammatory response in the brain-dead donor [38,58]. Furthermore, the use of C5a receptor antagonists has been effective in attenuating the extent of IRI in small animal models and limiting fibrogenic responses after acute kidney injury [39,78,104]. Currently, a clinical trial is being conducted to study the impact of eculizumab in the prevention of DGF in recipients of kidneys recovered from deceased donors (NCT01919346). The use of eculizumab for treatment of deceased donors prior to transplantation has yet to be explored.

## Conclusions

The multiple interactions between injury, immune response, and tissue repair are still a matter of intense study. It is now accepted that the complement system plays a central role in the pathogenesis of renal IRI and in the mechanisms leading to tissue damage in the deceased organ donor. Current evidence suggests that the formation of the terminal products of activation, such as MAC (C5b-9) and the generation of anaphylatoxins C3a and C5a, are responsible for triggering pro-inflammatory responses detrimental to ischemic tissue and antibody-mediated rejection. Further, a growing body of work suggests an important role in the modulation of adaptive immune responses in a wide variety of pathological conditions. More importantly, recent research indicates a role for complement in the regulation of tissue repair and the progression of fibrosis in models of acute kidney injury. These findings place the complement system as a centerpiece target in the elucidation of the precise mechanisms governing adequate (adaptive) and abnormal (maladaptive) tissue repair. However, the translation of therapies based on complement inhibition from the bench to bedside has been lacking and more research is required to fully understand the role of complement in the context of clinical IRI and transplantation. The development of targeted treatment strategies that lessen the need for immunosuppression of transplant recipients and that also have the ability to reduce inflammatory injury and tissue fibrosis is of paramount importance to maximize the limited organ donor pool and improve current transplant outcomes worldwide.

## Abbreviations

aHUS: Atypical hemolytic uremic syndrome; AMR: Antibody-mediated rejection; C1-INH: C1 inhibitor; C5aR: C5a receptors; CR1: Complement receptor 1; DAMP: Danger Associated Molecular Pattern; DGF: Delayed graft function; DSA: Donor specific antibodies; EndMT: Endothelial to mesenchymal transition; FDA: Food and drug administration; IRI: Ischemia-reperfusion injury; MAC: Membrane attack complex; MASP: MBL-associated proteins; MBL: Mannose-binding lectin; PGD: Primary graft dysfunction; PMNs: Polymorphonuclear neutrophils; ROS: Reactive oxygen species.

## Competing interests

LAF has received research funding from Pharming Technologies NV, Leiden, Netherlands. JSD and AJ declare that they have no competing interests.

## Authors' contributions

JSD, AJ, and LAF developed the review concept. JSD performed the literature review, created figures, and wrote the manuscript. All authors read and approved the final manuscript.

## References

[B1] Ricklin D Hajishengallis G Yang K Lambris JD Complement: a key system for immune surveillance and homeostasis Nat Immunol 2010 11 9 785 797 10.1038/ni.1923 20720586 PMC2924908

[B2] Ricklin D Lambris JD Complement in immune and inflammatory disorders: pathophysiological mechanisms J Immunol 2013 190 8 3831 3838 10.4049/jimmunol.1203487 23564577 PMC3623009

[B3] Zipfel PF Skerka C Complement regulators and inhibitory proteins Nat Rev Immunol 2009 9 10 729 740 19730437 10.1038/nri2620

[B4] Blom AM Villoutreix BO Dahlbäck B Complement inhibitor C4b-binding protein-friend or foe in the innate immune system? Mol Immunol 2004 40 18 1333 1346 10.1016/j.molimm.2003.12.002 15072852

[B5] Józsi M Zipfel PF Factor H family proteins and human diseases Trends Immunol 2008 29 8 380 387 10.1016/j.it.2008.04.008 18602340

[B6] Davis AE Biological effects of C1 inhibitor Drug News Perspect 2004 17 7 439 446 10.1358/dnp.2004.17.7.863703 15514703

[B7] Eltzschig HK Eckle T Ischemia and reperfusion–from mechanism to translation Nat Med 2011 17 11 1391 1401 10.1038/nm.2507 22064429 PMC3886192

[B8] Kono H Rock KL How dying cells alert the immune system to danger Nat Rev Immunol 2008 8 4 279 289 10.1038/nri2215 18340345 PMC2763408

[B9] Friedewald JJ Rabb H Inflammatory cells in ischemic acute renal failure Kidney Int 2004 66 2 486 491 10.1111/j.1523-1755.2004.761_3.x 15253694

[B10] Bonventre JV Zuk A Ischemic acute renal failure: an inflammatory disease? Kidney Int 2004 66 2 480 485 10.1111/j.1523-1755.2004.761_2.x 15253693

[B11] Diepenhorst GM van Gulik TM Hack CE Complement-mediated ischemia-reperfusion injury: lessons learned from animal and clinical studies Ann Surg 2009 249 6 889 899 10.1097/SLA.0b013e3181a38f45 19474697

[B12] Guo RF Ward PA Role of C5a in inflammatory responses Annu Rev Immunol 2005 23 821 852 10.1146/annurev.immunol.23.021704.115835 15771587

[B13] McCaughan JA O'Rourke DM Courtney AE The complement cascade in kidney disease: from sideline to center stage Am J Kidney Dis 2013 62 3 604 614 10.1053/j.ajkd.2012.12.033 23489674

[B14] Gasque P Complement: a unique innate immune sensor for danger signals Mol Immunol 2004 41 11 1089 1098 10.1016/j.molimm.2004.06.011 15476920

[B15] Zhou W Farrar CA Abe K Pratt JR Marsh JE Wang Y Stahl GL Sacks SH Predominant role for C5b-9 in renal ischemia/reperfusion injury J Clin Invest 2000 105 10 1363 1371 10.1172/JCI8621 10811844 PMC315463

[B16] Weiser MR Williams JP Moore FD Kobzik L Ma M Hechtman HB Carroll MC Reperfusion injury of ischemic skeletal muscle is mediated by natural antibody and complement J Exp Med 1996 183 5 2343 2348 10.1084/jem.183.5.2343 8642343 PMC2192547

[B17] Park P Haas M Cunningham PN Bao L Alexander JJ Quigg RJ Injury in renal ischemia-reperfusion is independent from immunoglobulins and T lymphocytes Am J Physiol Renal Physiol 2002 282 2 F352 F357 11788450 10.1152/ajprenal.00160.2001

[B18] Lin T Zhou W Farrar CA Hargreaves RE Sheerin NS Sacks SH Deficiency of C4 from donor or recipient mouse fails to prevent renal allograft rejection Am J Pathol 2006 168 4 1241 1248 10.2353/ajpath.2006.050360 16565498 PMC1606553

[B19] Thurman JM Ljubanovic D Edelstein CL Gilkeson GS Holers VM Lack of a functional alternative complement pathway ameliorates ischemic acute renal failure in mice J Immunol 2003 170 3 1517 1523 10.4049/jimmunol.170.3.1517 12538716

[B20] de Vries B Walter SJ Peutz-Kootstra CJ Wolfs TG van Heurn LW Buurman WA The mannose-binding lectin-pathway is involved in complement activation in the course of renal ischemia-reperfusion injury Am J Pathol 2004 165 5 1677 1688 10.1016/S0002-9440(10)63424-4 15509537 PMC1618654

[B21] Møller-Kristensen M Wang W Ruseva M Thiel S Nielsen S Takahashi K Shi L Ezekowitz A Jensenius JC Gadjeva M Mannan-binding lectin recognizes structures on ischaemic reperfused mouse kidneys and is implicated in tissue injury Scand J Immunol 2005 61 5 426 434 10.1111/j.1365-3083.2005.01591.x 15882434

[B22] Schwaeble WJ Lynch NJ Clark JE Marber M Samani NJ Ali YM Dudler T Parent B Lhotta K Wallis R Farrar CA Sacks S Lee H Zhang M Iwaki D Takahashi M Fujita T Tedford CE Stover CM Targeting of mannan-binding lectin-associated serine protease-2 confers protection from myocardial and gastrointestinal ischemia/reperfusion injury Proc Natl Acad Sci U S A 2011 108 18 7523 7528 10.1073/pnas.1101748108 21502512 PMC3088599

[B23] van der Pol P Schlagwein N van Gijlswijk DJ Berger SP Roos A Bajema IM de Boer HC de Fijter JW Stahl GL Daha MR van Kooten C Mannan-binding lectin mediates renal ischemia/reperfusion injury independent of complement activation Am J Transplant 2012 12 4 877 887 10.1111/j.1600-6143.2011.03887.x 22225993

[B24] Castellano G Melchiorre R Loverre A Ditonno P Montinaro V Rossini M Divella C Battaglia M Lucarelli G Annunziata G Palazzo S Selvaggi FP Staffieri F Crovace A Daha MR Mannesse M van Wetering S Paolo Schena F Grandaliano G Therapeutic targeting of classical and lectin pathways of complement protects from ischemia-reperfusion-induced renal damage Am J Pathol 2010 176 4 1648 1659 10.2353/ajpath.2010.090276 20150432 PMC2843457

[B25] Berger SP Roos A Mallat MJ Fujita T de Fijter JW Daha MR Association between mannose-binding lectin levels and graft survival in kidney transplantation Am J Transplant 2005 5 6 1361 1366 10.1111/j.1600-6143.2005.00841.x 15888042

[B26] Damman J Kok JL Snieder H Leuvenink HG van Goor H Hillebrands JL van Dijk MC Hepkema BG Reznichenko A van den Born J de Borst MH Bakker SJ Navis GJ Ploeg RJ Seelen MA Lectin complement pathway gene profile of the donor and recipient does not influence graft outcome after kidney transplantation Mol Immunol 2012 50 1–2 1 8 22173059 10.1016/j.molimm.2011.11.009

[B27] Bay JT Sørensen SS Hansen JM Madsen HO Garred P Low mannose-binding lectin serum levels are associated with reduced kidney graft survival Kidney Int 2013 83 2 264 271 10.1038/ki.2012.373 23172101

[B28] Heijnen BH Straatsburg IH Padilla ND Van Mierlo GJ Hack CE Van Gulik TM Inhibition of classical complement activation attenuates liver ischaemia and reperfusion injury in a rat model Clin Exp Immunol 2006 143 1 15 23 10.1111/j.1365-2249.2005.02958.x 16367929 PMC1809558

[B29] Straatsburg IH Boermeester MA Wolbink GJ van Gulik TM Gouma DJ Frederiks WM Hack CE Complement activation induced by ischemia-reperfusion in humans: a study in patients undergoing partial hepatectomy J Hepatol 2000 32 5 783 791 10.1016/S0168-8278(00)80247-0 10845665

[B30] Williams JP Pechet TT Weiser MR Reid R Kobzik L Moore FD Carroll MC Hechtman HB Intestinal reperfusion injury is mediated by IgM and complement J Appl Physiol (1985) 1999 86 3 938 942 10066708 10.1152/jappl.1999.86.3.938

[B31] Hill JH Ward PA The phlogistic role of C3 leukotactic fragments in myocardial infarcts of rats J Exp Med 1971 133 4 885 900 10.1084/jem.133.4.885 4993831 PMC2138969

[B32] Jordan JE Montalto MC Stahl GL Inhibition of mannose-binding lectin reduces postischemic myocardial reperfusion injury Circulation 2001 104 12 1413 1418 10.1161/hc3601.095578 11560858

[B33] Sacks SH Complement fragments C3a and C5a: the salt and pepper of the immune response Eur J Immunol 2010 40 3 668 670 10.1002/eji.201040355 20186746

[B34] Zhou W The new face of anaphylatoxins in immune regulation Immunobiology 2012 217 2 225 234 10.1016/j.imbio.2011.07.016 21856033

[B35] van der Touw W Cravedi P Kwan WH Paz-Artal E Merad M Heeger PS Cutting edge: receptors for C3a and C5a modulate stability of alloantigen-reactive induced regulatory T cells J Immunol 2013 190 12 5921 5925 10.4049/jimmunol.1300847 23690475 PMC3679341

[B36] Cravedi P Leventhal J Lakhani P Ward SC Donovan MJ Heeger PS Immune cell-derived C3a and C5a costimulate human T cell alloimmunity Am J Transplant 2013 13 10 2530 2539 10.1111/ajt.12405 24033923 PMC3809075

[B37] Peng Q Li K Smyth LA Xing G Wang N Meader L Lu B Sacks SH Zhou W C3a and C5a promote renal ischemia-reperfusion injury J Am Soc Nephrol 2012 23 9 1474 1485 10.1681/ASN.2011111072 22797180 PMC3431410

[B38] de Vries B Köhl J Leclercq WK Wolfs TG van Bijnen AA Heeringa P Buurman WA Complement factor C5a mediates renal ischemia-reperfusion injury independent from neutrophils J Immunol 2003 170 7 3883 3889 10.4049/jimmunol.170.7.3883 12646657

[B39] Arumugam TV Shiels IA Strachan AJ Abbenante G Fairlie DP Taylor SM A small molecule C5a receptor antagonist protects kidneys from ischemia/reperfusion injury in rats Kidney Int 2003 63 1 134 142 10.1046/j.1523-1755.2003.00737.x 12472776

[B40] Zheng X Zhang X Feng B Sun H Suzuki M Ichim T Kubo N Wong A Min LR Budohn ME Garcia B Jevnikar AM Min WP Gene silencing of complement C5a receptor using siRNA for preventing ischemia/reperfusion injury Am J Pathol 2008 173 4 973 980 10.2353/ajpath.2008.080103 18772341 PMC2543066

[B41] Djamali A Muth BL Ellis TM Mohamed M Fernandez LA Miller KM Bellingham JM Odorico JS Mezrich JD Pirsch JD D'Alessandro TM Vidyasagar V Hofmann RM Torrealba JR Kaufman DB Foley DP Increased C4d in post-reperfusion biopsies and increased donor specific antibodies at one-week post transplant are risk factors for acute rejection in mild to moderately sensitized kidney transplant recipients Kidney Int 2013 83 6 1185 1192 10.1038/ki.2013.44 23447068 PMC3672254

[B42] Stegall MD Diwan T Raghavaiah S Cornell LD Burns J Dean PG Cosio FG Gandhi MJ Kremers W Gloor JM Terminal complement inhibition decreases antibody-mediated rejection in sensitized renal transplant recipients Am J Transplant 2011 11 11 2405 2413 10.1111/j.1600-6143.2011.03757.x 21942930

[B43] Lefaucheur C Loupy A Hill GS Andrade J Nochy D Antoine C Gautreau C Charron D Glotz D Suberbielle-Boissel C Preexisting donor-specific HLA antibodies predict outcome in kidney transplantation J Am Soc Nephrol 2010 21 8 1398 1406 10.1681/ASN.2009101065 20634297 PMC2938596

[B44] Lerut E Naesens M Kuypers DR Vanrenterghem Y Van Damme B Subclinical peritubular capillaritis at 3 months is associated with chronic rejection at 1 year Transplantation 2007 83 11 1416 1422 10.1097/01.tp.0000266676.10550.70 17565313

[B45] Dean PG Park WD Cornell LD Gloor JM Stegall MD Intragraft gene expression in positive crossmatch kidney allografts: ongoing inflammation mediates chronic antibody-mediated injury Am J Transplant 2012 12 6 1551 1563 10.1111/j.1600-6143.2011.03964.x 22335458

[B46] Floerchinger B Oberhuber R Tullius SG Effects of brain death on organ quality and transplant outcome Transplant Rev (Orlando) 2012 26 2 54 59 10.1016/j.trre.2011.10.001 22459036

[B47] Danobeitia JS Sperger JM Hanson MS Park EE Chlebeck PJ Roenneburg DA Sears ML Connor JX Schwarznau A Fernandez LA Early activation of the inflammatory response in the liver of brain-dead non-human primates J Surg Res 2012 176 2 639 648 10.1016/j.jss.2011.10.042 22440934 PMC7087484

[B48] van der Hoeven J Molema G Ter Horst G Freund R Wiersema J van Schilfgaarde R Leuvenink H Ploeg R Relationship between duration of brain death and hemodynamic (in)stability on progressive dysfunction and increased immunologic activation of donor kidneys Kidney Int 2003 64 5 1874 1882 10.1046/j.1523-1755.2003.00272.x 14531823

[B49] Damman J Schuurs TA Ploeg RJ Seelen MA Complement and renal transplantation: from donor to recipient Transplantation 2008 85 7 923 927 10.1097/TP.0b013e3181683cf5 18408568

[B50] Kusaka M Pratschke J Wilhelm MJ Ziai F Zandi-Nejad K Mackenzie HS Hancock WW Tilney NL Activation of inflammatory mediators in rat renal isografts by donor brain death Transplantation 2000 69 3 405 410 10.1097/00007890-200002150-00017 10706051

[B51] Pratt JR Abe K Miyazaki M Zhou W Sacks SH In situ localization of C3 synthesis in experimental acute renal allograft rejection Am J Pathol 2000 157 3 825 831 10.1016/S0002-9440(10)64596-8 10980122 PMC1885894

[B52] Pratt JR Basheer SA Sacks SH Local synthesis of complement component C3 regulates acute renal transplant rejection Nat Med 2002 8 6 582 587 10.1038/nm0602-582 12042808

[B53] Damman J Nijboer WN Schuurs TA Leuvenink HG Morariu AM Tullius SG van Goor H Ploeg RJ Seelen MA Local renal complement C3 induction by donor brain death is associated with reduced renal allograft function after transplantation Nephrol Dial Transplant 2011 26 7 2345 2354 10.1093/ndt/gfq717 21127132

[B54] Atkinson C Varela JC Tomlinson S Complement-dependent inflammation and injury in a murine model of brain dead donor hearts Circ Res 2009 105 11 1094 1101 10.1161/CIRCRESAHA.109.194977 19815824 PMC2783176

[B55] Damman J Hoeger S Boneschansker L Theruvath A Waldherr R Leuvenink HG Ploeg RJ Yard BA Seelen MA Targeting complement activation in brain-dead donors improves renal function after transplantation Transpl Immunol 2011 24 4 233 237 10.1016/j.trim.2011.03.001 21440065

[B56] Naesens M Li L Ying L Sansanwal P Sigdel TK Hsieh SC Kambham N Lerut E Salvatierra O Butte AJ Sarwal MM Expression of complement components differs between kidney allografts from living and deceased donors J Am Soc Nephrol 2009 20 8 1839 1851 10.1681/ASN.2008111145 19443638 PMC2723986

[B57] Damman J Seelen MA Moers C Daha MR Rahmel A Leuvenink HG Paul A Pirenne J Ploeg RJ Systemic complement activation in deceased donors is associated with acute rejection after renal transplantation in the recipient Transplantation 2011 92 2 163 169 10.1097/TP.0b013e318222c9a0 21677599

[B58] van Werkhoven MB Damman J van Dijk MC Daha MR de Jong IJ Leliveld A Krikke C Leuvenink HG van Goor H van Son WJ Olinga P Hillebrands JL Seelen MA Complement mediated renal inflammation induced by donor brain death: role of renal C5a-C5aR interaction Am J Transplant 2013 13 4 875 882 10.1111/ajt.12130 23398742

[B59] Błogowski W Dołęgowska B Sałata D Budkowska M Domański L Starzyńska T Clinical analysis of perioperative complement activity during ischemia/reperfusion injury following renal transplantation Clin J Am Soc Nephrol 2012 7 11 1843 1851 10.2215/CJN.02200312 22904122 PMC3488944

[B60] de Vries DK van der Pol P van Anken GE van Gijlswijk DJ Damman J Lindeman JH Reinders ME Schaapherder AF Kooten C Acute but transient release of terminal complement complex after reperfusion in clinical kidney transplantation Transplantation 2013 95 6 816 820 10.1097/TP.0b013e31827e31c9 23348894

[B61] Mastellos DC Deangelis RA Lambris JD Complement-triggered pathways orchestrate regenerative responses throughout phylogenesis Semin Immunol 2013 25 1 29 38 10.1016/j.smim.2013.04.002 23684626 PMC3920450

[B62] Vasel M Rutz R Bersch C Feick P Singer MV Kirschfink M Nakchbandi IA Complement activation correlates with liver necrosis and fibrosis in chronic hepatitis C Clin Immunol 2014 150 2 149 156 10.1016/j.clim.2013.11.014 24412908

[B63] Khan MA Nicolls MR Complement-mediated microvascular injury leads to chronic rejection Adv Exp Med Biol 2013 735 233 246 10.1007/978-1-4614-4118-2_16 23402031 PMC4015512

[B64] Khan MA Maasch C Vater A Klussmann S Morser J Leung LL Atkinson C Tomlinson S Heeger PS Nicolls MR Targeting complement component 5a promotes vascular integrity and limits airway remodeling Proc Natl Acad Sci U S A 2013 110 15 6061 6066 10.1073/pnas.1217991110 23530212 PMC3625314

[B65] DeAngelis RA Markiewski MM Kourtzelis I Rafail S Syriga M Sandor A Maurya MR Gupta S Subramaniam S Lambris JD A complement-IL-4 regulatory circuit controls liver regeneration J Immunol 2012 188 2 641 648 10.4049/jimmunol.1101925 22184721 PMC3253144

[B66] Strey CW Markiewski M Mastellos D Tudoran R Spruce LA Greenbaum LE Lambris JD The proinflammatory mediators C3a and C5a are essential for liver regeneration J Exp Med 2003 198 6 913 923 10.1084/jem.20030374 12975457 PMC2194207

[B67] He S Atkinson C Qiao F Cianflone K Chen X Tomlinson S A complement-dependent balance between hepatic ischemia/reperfusion injury and liver regeneration in mice J Clin Invest 2009 119 8 2304 2316 19620784 10.1172/JCI38289 PMC2719951

[B68] Su Z Wang X Gao X Liu Y Pan C Hu H Beyer RP Shi M Zhou J Zhang J Serra AL Wüthrich RP Mei C Excessive activation of the alternative complement pathway in autosomal dominant polycystic kidney disease J Intern Med 2014 In press 10.1111/joim.12214 24494798

[B69] Hepburn NJ Ruseva MM Harris CL Morgan BP Complement, roles in renal disease and modulation for therapy Clin Nephrol 2008 70 5 357 376 19000536 10.5414/cnp70357

[B70] Mele C Remuzzi G Noris M Hemolytic uremic syndrome Semin Immunopathol 2014 In press 10.1007/s00281-014-0416-x 24526222

[B71] Sacks SH Zhou W The role of complement in the early immune response to transplantation Nat Rev Immunol 2012 12 6 431 442 10.1038/nri3225 22627861

[B72] Bonventre JV Yang L Cellular pathophysiology of ischemic acute kidney injury J Clin Invest 2011 121 11 4210 4221 10.1172/JCI45161 22045571 PMC3204829

[B73] Ko GJ Boo CS Jo SK Cho WY Kim HK Macrophages contribute to the development of renal fibrosis following ischaemia/reperfusion-induced acute kidney injury Nephrol Dial Transplant 2008 23 3 842 852 17984109 10.1093/ndt/gfm694

[B74] Sutton TA Mang HE Campos SB Sandoval RM Yoder MC Molitoris BA Injury of the renal microvascular endothelium alters barrier function after ischemia Am J Physiol Renal Physiol 2003 285 2 F191 F198 12684225 10.1152/ajprenal.00042.2003

[B75] Basile DP The endothelial cell in ischemic acute kidney injury: implications for acute and chronic function Kidney Int 2007 72 2 151 156 10.1038/sj.ki.5002312 17495858

[B76] Koo DD Welsh KI West NE Channon KM Penington AJ Roake JA Morris PJ Fuggle SV Endothelial cell protection against ischemia/reperfusion injury by lecithinized superoxide dismutase Kidney Int 2001 60 2 786 796 10.1046/j.1523-1755.2001.060002786.x 11473663

[B77] Tipping PG Huang XR Berndt MC Holdsworth SR A role for P selectin in complement-independent neutrophil-mediated glomerular injury Kidney Int 1994 46 1 79 88 10.1038/ki.1994.246 7523757

[B78] Boor P Konieczny A Villa L Schult AL Bücher E Rong S Kunter U van Roeyen CR Polakowski T Hawlisch H Hillebrandt S Lammert F Eitner F Floege J Ostendorf T Complement C5 mediates experimental tubulointerstitial fibrosis J Am Soc Nephrol 2007 18 5 1508 1515 10.1681/ASN.2006121343 17389734

[B79] Bao L Wang Y Haas M Quigg RJ Distinct roles for C3a and C5a in complement-induced tubulointerstitial injury Kidney Int 2011 80 5 524 534 10.1038/ki.2011.158 21677637

[B80] Guerrot D Dussaule J-C Kavvadas P Boffa J-J Chadjichristos CE Chatziantoniou C Progression of renal fibrosis: the underestimated role of endothelial alterations Fibrogenesis Tissue Repair 2012 5 Suppl 1 S15 10.1186/1755-1536-5-S1-S15 23259724 PMC3368764

[B81] Basile DP Friedrich JL Spahic J Knipe N Mang H Leonard EC Changizi-Ashtiyani S Bacallao RL Molitoris BA Sutton TA Impaired endothelial proliferation and mesenchymal transition contribute to vascular rarefaction following acute kidney injury Am J Physiol Renal Physiol 2011 300 3 F721 F733 10.1152/ajprenal.00546.2010 21123492 PMC3064142

[B82] Curci C Castellano G Stasi A Divella C Loverre A Gigante M Simone S Cariello M Montinaro V Lucarelli G Ditonno P Battaglia M Crovace A Staffieri F Oortwijn B van Amersfoort E Gesualdo L Grandaliano G Endothelial-to-mesenchymal transition and renal fibrosis in ischaemia/reperfusion injury are mediated by complement anaphylatoxins and Akt pathway Nephrol Dial Transplant 2014 29 4 799 808 10.1093/ndt/gft516 24463188

[B83] Wan JX Fukuda N Endo M Tahira Y Yao EH Matsuda H Ueno T Matsumoto K Complement 3 is involved in changing the phenotype of human glomerular mesangial cells J Cell Physiol 2007 213 2 495 501 10.1002/jcp.21129 17520688

[B84] Rangan GK Pippin JW Couser WG C5b-9 regulates peritubular myofibroblast accumulation in experimental focal segmental glomerulosclerosis Kidney Int 2004 66 5 1838 1848 10.1111/j.1523-1755.2004.00957.x 15496154

[B85] Ricardo SD van Goor H Eddy AA Macrophage diversity in renal injury and repair J Clin Invest 2008 118 11 3522 3530 10.1172/JCI36150 18982158 PMC2575702

[B86] Mannon RB Macrophages: contributors to allograft dysfunction, repair, or innocent bystanders? Curr Opin Organ Transplant 2012 17 1 20 25 10.1097/MOT.0b013e32834ee5b6 22157320 PMC3319132

[B87] Li L Huang L Sung SS Vergis AL Rosin DL Rose CE Jr Lobo PI Okusa MD The chemokine receptors CCR2 and CX3CR1 mediate monocyte/macrophage trafficking in kidney ischemia-reperfusion injury Kidney Inter 2008 74 12 1526 1537 10.1038/ki.2008.500 PMC2652647 18843253

[B88] Phieler J Chung K-J Chatzigeorgiou A Ameln AK-v Garcia-Martin R Sprott D Moisidou M Tzanavari T Ludwig B Baraban E Ehrhart-Bornstein M Bornstein SR Mziaut H Solimena M Karalis KP Economopoulou M Lambris JD Chavakis T The complement anaphylatoxin C5a receptor contributes to obese adipose tissue inflammation and insulin resistance J Immunol 2013 191 8 4367 4374 10.4049/jimmunol.1300038 24043887 PMC3817864

[B89] Bedard K Krause KH The NOX family of ROS-generating NADPH oxidases: physiology and pathophysiology Physiol Rev 2007 87 1 245 313 10.1152/physrev.00044.2005 17237347

[B90] Gill PS Wilcox CS NADPH oxidases in the kidney Antioxid Redox Signal 2006 8 9–10 1597 1607 16987014 10.1089/ars.2006.8.1597

[B91] Gardinali M Padalino P Vesconi S Calcagno A Ciappellano S Conciato L Chiara O Agostoni A Nespoli A Complement activation and polymorphonuclear neutrophil leukocyte elastase in sepsis. Correlation with severity of disease Arch Surg 1992 127 10 1219 1224 10.1001/archsurg.1992.01420100077014 1417490

[B92] Igonin AA Protsenko DN Galstyan GM Vlasenko AV Khachatryan NN Nekhaev IV Shlyapnikov SA Lazareva NB Herscu P C1-esterase inhibitor infusion increases survival rates for patients with sepsis* Crit Care Med 2012 40 3 770 777 10.1097/CCM.0b013e318236edb8 22080632

[B93] Djamali A Reese S Hafez O Vidyasagar A Jacobson L Swain W Kolehmainen C Huang L Wilson NA Torrealba JR Nox2 is a mediator of chronic CsA nephrotoxicity Am J Transplant 2012 12 8 1997 2007 10.1111/j.1600-6143.2012.04081.x 22568654 PMC3409317

[B94] Djamali A Vidyasagar A Adulla M Hullett D Reese S Nox-2 is a modulator of fibrogenesis in kidney allografts Am J Transplant 2009 9 1 74 82 18976289 10.1111/j.1600-6143.2008.02463.x PMC3572864

[B95] Davis AE Lu F Mejia P C1 inhibitor, a multi-functional serine protease inhibitor Thromb Haemost 2010 104 5 886 893 10.1160/TH10-01-0073 20806108

[B96] Frank MM Recombinant and plasma-purified human c1 inhibitor for the treatment of hereditary angioedema World Allergy Organ J 2010 3 9 Suppl S29 S33 23282867 10.1097/1939-4551-3-S3-S29 PMC3666149

[B97] Lu F Chauhan AK Fernandes SM Walsh MT Wagner DD Davis AE The effect of C1 inhibitor on intestinal ischemia and reperfusion injury Am J Physiol Gastrointest Liver Physiol 2008 295 5 G1042 G1049 10.1152/ajpgi.90460.2008 18787060

[B98] Lu F Fernandes SM Davis AE The effect of C1 inhibitor on myocardial ischemia and reperfusion injury Cardiovasc Pathol 2013 22 1 75 80 10.1016/j.carpath.2012.05.003 22705194 PMC3449044

[B99] Saidi RF Rajeshkumar B Shariftabrizi A Dresser K Walter O Human C1 inhibitor attenuates liver ischemia-reperfusion injury and promotes liver regeneration J Surg Res 2014 187 2 660 666 10.1016/j.jss.2013.09.009 24433870

[B100] Gesuete R Storini C Fantin A Stravalaci M Zanier ER Orsini F Vietsch H Mannesse ML Ziere B Gobbi M De Simoni MG Recombinant C1 inhibitor in brain ischemic injury Ann Neurol 2009 66 3 332 342 10.1002/ana.21740 19798727

[B101] Tillou X Poirier N Le Bas-Bernardet S Hervouet J Minault D Renaudin K Vistoli F Karam G Daha M Soulillou JP Blancho G Recombinant human C1-inhibitor prevents acute antibody-mediated rejection in alloimmunized baboons Kidney Int 2010 78 2 152 159 10.1038/ki.2010.75 20336054

[B102] Sommer W Tudorache I Kühn C Avsar M Salman J Ius F Gras C Weber P Welte T Gottlieb J Haverich A Warnecke G C1-esterase-inhibitor for primary graft dysfunction in lung transplantation Transplantation 2014 97 11 1185 1191 10.1097/TP.0000000000000034 24573112

[B103] Barnett AN Asgari E Chowdhury P Sacks SH Dorling A Mamode N The use of eculizumab in renal transplantation Clin Transplant 2013 27 3 E216 E229 10.1111/ctr.12102 23516966

[B104] De Vries B Matthijsen RA Wolfs TG Van Bijnen AA Heeringa P Buurman WA Inhibition of complement factor C5 protects against renal ischemia-reperfusion injury: inhibition of late apoptosis and inflammation Transplantation 2003 75 3 375 382 10.1097/01.TP.0000044455.05584.2A 12589162

